# Effect of Nitrogen Sources on Diatoms Growth and Nutritional Value for Enhancing *Litopenaeus vannamei* Larval Performance

**DOI:** 10.3390/ani15040466

**Published:** 2025-02-07

**Authors:** Reham A. Abdelhay, Mohammad S. El-Mor, Mohammed A. M. Salem, Adham A. Al-Sagheer, Yasmina M. Abd-Elhakim, Bayan A. Hassan, Hossam A. M. Mounes

**Affiliations:** 1 Limnology Department, Central Laboratory for Aquaculture Research, Agricultural Research Center, Abassa, Abu Hammad 44662, Egypt; hmoans80@hotmail.com; 2 Marine Science Department, Faculty of Science, Suez Canal University, Ismailia 41522, Egypt; 3 Animal Production Department, Faculty of Agriculture, Zagazig University, Zagazig 44511, Egypt; 4 Department of Forensic Medicine and Toxicology, Faculty of Veterinary Medicine, Zagazig University, Zagazig 44519, Egypt; 5 Pharmacology Department, Faculty of Pharmacy, Future University, Cairo 11835, Egypt; bayan.saffaf@fue.edu.eg

**Keywords:** diatom growth, *Chaetocerus calcitrans*, *Thalassiosira weissflogii*, *Litopenaeus vannamei* larvae, nitrogen sources, live food diets

## Abstract

This study investigated the impact of different nitrogen sources on the growth and biochemical composition of two diatom species, *Chaetoceros calcitrans* and *Thalassiosira weissflogii*, and evaluated their use as live feed for *Litopenaeus vannamei* larvae. Diatoms were cultured in a Conway medium supplemented with four nitrogen sources: potassium nitrate (control), urea, ammonium sulfate, and ammonium nitrate. In a separate experiment, white-leg shrimp larvae (300 larvae/L) at stage Nauplius 6 were fed diets consisting of *C. calcitrans*, *T. weissflogii*, or a combination of both diatoms under controlled conditions. The results indicated that urea, ammonium nitrate, and ammonium sulfate significantly enhanced the growth and nutrient composition of *C. calcitrans* and *T. weissflogii* compared to the control (potassium nitrate). In *C. calcitrans*, ammonium nitrate significantly increased protein and lipid contents, while carbohydrate levels were the highest in the control. Similarly, urea and ammonium sulfate treatments yielded the highest lipid levels, whereas the control exhibited the lowest. For *T. weissflogii*, the control achieved the highest cell count on day 4, but ammonium nitrate significantly improved protein and lipid contents while reducing carbohydrate levels. A mixed diet of *C. calcitrans* and *T. weissflogii* significantly enhanced growth performance and reduced mortality rates in *L. vannamei* larvae compared to single-species diets. In conclusion, the findings indicated that ammonium nitrate was an efficient nitrogen source for enhancing diatom growth. Additionally, combining *C. calcitrans* and *T. weissflogii* as a diet improved growth and survival of *L. vannamei* larvae, offering practical implications for aquaculture.

## Simple Summary

Shrimp larvae, particularly *Litopenaeus vannamei*, rely heavily on high-quality, nutrient-rich diets to support their growth during the early stages of life. Diatoms, as rich sources of essential fatty acids, proteins, and other bioactive compounds, play a crucial role in larval nutrition. This study evaluated the effects of four nitrogen sources on the growth and chemical composition of two diatom species, *Chaetoceros calcitrans* and *Thalassiosira weissflogii*, and evaluated their use as live feed for *Litopenaeus vannamei* larvae. The results indicated that lower-cost nitrogen sources, such as urea, ammonium sulfate, and ammonium nitrate, significantly enhanced the growth and biomass production of both diatoms compared with the control (potassium nitrate). Both *C. calcitrans* and *T. weissflogii* exhibited improved protein and lipid content when cultured with lower cost nitrogen source. These sources also led to a notable reduction in carbohydrate content, optimizing the nutritional profile of the diatoms. Additionally, feeding *L. vannamei* larvae a mixed diet of *C. calcitrans* and *T. weissflogii* leads to significant improvements, including lower mortality rates and higher growth performance, compared to diets containing a single diatom species. In conclusion, urea, ammonium sulfate, and ammonium nitrate as nitrogen sources boost the growth and improve the nutritional quality of the diatom species *C. calcitrans* and *T. weissflogii*-based diets for shrimp larvae, providing a cost-effective solution for shrimp aquaculture.

## 1. Introduction

Aquaculture is one of the fastest-growing sectors in global food production. Global aquaculture and fisheries production reached nearly 223 million tonnes in 2022, marking a 4.4% increase from 2020. This total includes 185.4 million tonnes of aquatic animals and 37.8 million tonnes of algae [1]. Aquaculture plays a crucial role in meeting the increasing demand for protein-rich seafood and contributing significantly to global food security and economic development [2,3,4]. Among the various aquaculture sectors, shrimp farming has experienced rapid growth in recent years, especially in developing countries, where it has become a major source of both nutrition and income [5,6,7,8]. *Litopenaeus vannamei* is the preferred shrimp species due to its cultural characteristics and consumer acceptance [9]. The global production of *L. vannamei* grew from 155,000 tonnes in 2000 to 5.8 million tonnes in 2020 [10]. Recent advancements in shrimp aquaculture, particularly in feed innovations, breeding programs, and disease control, have contributed to this remarkable growth [11]. However, challenges such as escalating production costs, environmental sustainability, and reliance on commercial feeds necessitate further research into sustainable solutions. Feed ingredients represent the most significant variable cost in shrimp production, serving as a vital source of nutrients but also contributing to biological waste [12,13].

Commercial shrimp hatcheries rely on balanced feeds and nutritional supplements for larval growth, particularly in the early stages. Despite advances in formulated feeds, live feed remains essential, especially for Zoea larvae [14,15]. Among live feeds, phytoplankton, particularly diatoms, play a crucial role due to their rich nutritional composition, including proteins, lipids, and polyunsaturated fatty acids, which are vital for shrimp larval development and survival [16,17,18]. For instance, Bhattacharjya et al. [19] demonstrated that diatoms not only improve shrimp health and survival but also enhance water quality by removing eutrophic nutrients, promoting sustainable aquaculture practices.

Diatoms such as *Chaetoceros* spp. and *Thalassiosira weissflogii* are widely utilized as live feeds due to their rapid growth rates, rich nutritional content, and optimal fatty acid composition [20]. Tam et al. [21] highlighted the successful cultivation of *Thalassiosira weissflogii* as live feed for white-leg shrimp, demonstrating significant improvements in larval survival, growth, and metamorphosis rates, alongside enhanced nutritional profiles in shrimp flesh. Also, Bhattacharjya et al. [22] emphasized that diatoms can be cultivated in aquaculture wastewater, effectively utilizing excess nutrients while improving water quality by removing nitrogen and phosphorus. Diatoms must be of an appropriate size for ingestion, e.g., from 1 to 15 μm for filter feeders; 10 to 100 μm for grazers and readily digested [23,24]. Accurate identification of diatoms at the species level is essential in aquaculture studies and water quality management. Molecular techniques, such as DNA barcoding, are crucial for the accurate identification of diatom species, successfully eliminating the shortcomings of conventional morphological methods that depend on siliceous frustules [25].

Sufficient supplementation of nutrients for the growth of microalgae is a key step in producing a bulk quantity of high-quality microalgae biomass [26,27]. Nitrogen is a critical macronutrient for diatom growth, influencing the synthesis of proteins, chlorophylls, and other essential cellular components [28]. Different nitrogen sources, such as ammonium sulfate and nitrate-based compounds, may impact diatom growth and biochemical composition differently, potentially offering cost-effective alternatives to traditional feed formulations. Ammonium-based sources are directly assimilated into amino acids, while nitrate-based sources require reduction before utilization [29]. This difference in assimilation processes can significantly impact diatom growth and biochemical composition. Research indicated that ammonium generally promotes higher protein content, while nitrate tends to enhance lipid accumulation. For instance, when cultured with ammonium as the nitrogen source, *Amphora coffeaeformis* exhibited a protein content of 44.82% by dry weight and a lipid content of 18.59%. In contrast, nitrate cultures resulted in a higher lipid content of 29.78% but a lower protein content of 38.77% [28]. In both outdoor and indoor microalgae cultures for commercial aquaculture, microalgae growing mediums are frequently prepared using agricultural fertilizers, the majority of which are obtained from mineral sources. Pure-strain microalgae, however, require a more exacting nutritional composition in their growing media. The production of microalgae monoculture on a laboratory scale often uses specialized media formulations, such as Conway and f/2 media [28,30].

Although microalgae are commonly used as live feed in aquaculture, the combined feeding effect of *Chaetoceros calcitrans* and *Thalassiosira weissflogii* on shrimp larvae growth, survival, and feeding efficiency has not been previously studied. This research fills that gap by exploring how the nutritional quality of these diatoms, cultivated with cost-effective nitrogen sources (urea: 0.30 US$/kg, ammonium sulfate: 0.20 US$/kg, and ammonium nitrate: 0.30 US$/kg), influences shrimp larvae performance, offering practical alternatives to the expensive control (potassium nitrate: 100 US$/kg). The objective of the present study was to find out the effect of different nitrogen sources suitable for the production of two diatom species regarding growth and biochemical composition. And carry out the efficacy of microalgal species, such as *Chaetoceros calcitrans* and *Thalassiosira weissflogii*, on shrimp growth, survival, and feeding rate. Additionally, gene identification using *ITS-2* and *rbcL* sequences was performed to confirm the species of the diatoms.

## 2. Materials and Methods

### 2.1. Microalgae Culture

#### 2.1.1. Strain and Identification

**Figure 1:**
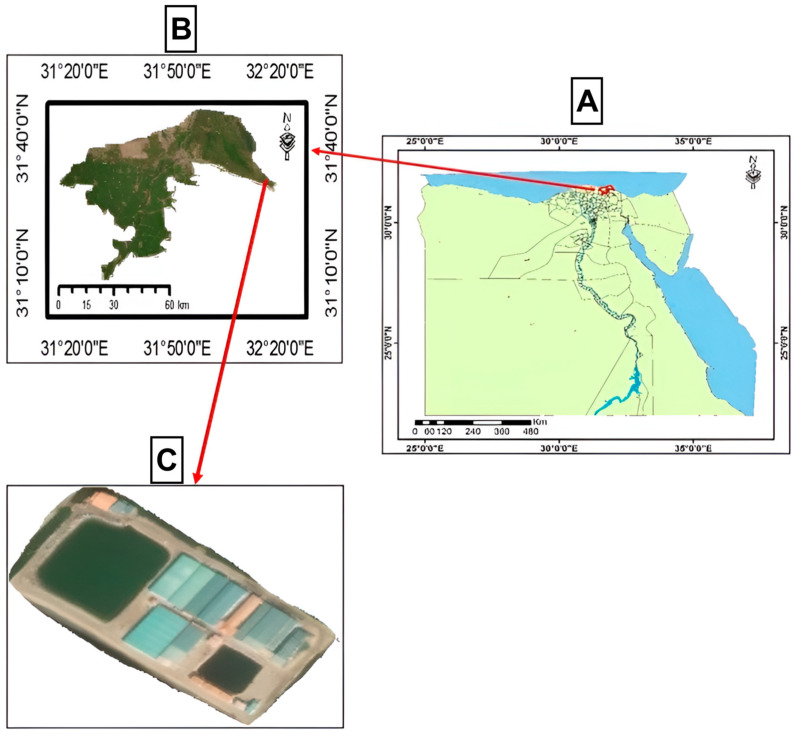
The study area at El-Ekhlas hatchery, located in Damietta Governorate near the Mediterranean Sea. (**A**) Map of Egypt, (**B**) Damietta Governorate, and (**C**) El-Ekhlas hatchery. Maps were generated using ArcGIS 10.8 software (Environmental Systems Research Institute, Redlands, CA, USA).

Diatoms of the genera *Chaetoceros* and *Thalassiosira* were obtained from the El-Ekhlas hatchery, located in the Damietta Governorate, Mediterranean Sea (Figure 1). Morphological and molecular identifications of the diatom samples were performed after collection to confirm their species before inclusion in the experiments. Molecular identification of microalgae samples was performed using an internal transcribed spacer (*ITS-2*) and ribulose-1,5-bisphosphate carboxylase/oxygenase large subunit (*rbcL*) gene markers.

The Conway medium supplemented with silicate was used for the diatom culture following the method described by Walne [31]. To prepare 1 L of the Conway medium, the following components were added to 1 L of distilled water: 1 mL of solution A (containing 116 g of KNO_3_, 45 g of Na_2_EDTA, 33.6 g of H_3_BO_3_, 20 g of NaH_2_PO_4_, 3.15 g of FeCl_3_, and 0.36 g of MnCl_2_.4H_2_O per liter), 1 mL of solution B (containing 50 g of Na_2_SiO_3_.9H_2_O per 100 mL) and 1 mL of trace metal solution (containing 2.01 g of ZnCl_2_, 9.8 g of CoCl_2_.6H_2_O, 6.3 g of (NH_4_)_6_Mo_7_O_24_.4H_2_O, and 2 g of CuSO_4_.5H_2_O per 100 mL). After autoclaving and cooling the medium, 0.5 mL of the vitamin solution was added. The vitamin solution contained 0.01 g of biotin (vitamin H), 0.02 g of thiamine HCl (vitamin B_1_), and 0.01 g of cyanocobalamin (vitamin B_12_) per 100 mL.

#### 2.1.2. Morphological Identification of Diatoms

Pure diatom samples of the genera *Chaetoceros* and *Thalassiosira* were collected and fixed using Lugol’s iodine solution to preserve the diatoms. To concentrate the diatom cells, the samples were centrifuged at 3000 rpm for 10 min. Following centrifugation, the concentrated diatom cells were retained at the bottom of the tube. For morphological identification, microscope slides were prepared by placing a drop of the concentrated sample onto a clean glass slide, which was then covered with a coverslip to avoid contamination and the formation of air bubbles. Observations were conducted using an optical light microscope equipped with 100× and 40× oil immersion lenses. The intricate morphology of the diatom species was examined following the guidelines of Hasle and Syvertsen [32].

#### 2.1.3. Molecular Identification of Microalgal Species

Molecular identification of microalgae samples was performed using *ITS-2* and *rbcL* gene markers. DNA was extracted from the diatom samples using the Algae DNA Isolation Kit (Catalog Number: NA2012-01, Attogene, Austin, TX, USA), which is specifically designed for algae. The kit utilizes proprietary magnetic beads and a specialized buffer to ensure high-quality DNA suitable for PCR amplification. The purified DNA was eluted with elution buffer (EL) and stored at −20 °C. Sequences of the *ITS-2* and *rbcL* genes were retrieved from the NCBI database. Primer sequences are listed in Table 1. Multiple sequence alignment was performed for each gene to identify conserved regions. PCR amplification was carried out using the following reaction mixture: 2 µL of the DNA template, 1 µL of each primer (10 pM), 1 U of the Taq DNA polymerase (Thermo Scientific), and other reagents to a final volume of 20 µL. The PCR program involved an initial denaturation at 94 °C for 5 min, followed by 35 cycles of denaturation at 94 °C for 40 s, annealing at 59 °C (for *ITS*) and 55 °C (for *rbcL*) for 40 s, and extension at 72 °C for 45 s, with a final extension step at 72 °C for 10 min. PCR products were confirmed by 1% agarose gel electrophoresis. The PCR products were sequenced using bidirectional sequencing or cloned into the PMD 18-T vector (Takara) for sequencing. Phylogenetic relationships for the *ITS-2* and *rbcL* gene sequences were inferred using Bayesian Inference (BI) with MrBayes version 3.2 [33]. Sequences were aligned with reference sequences from the NCBI GenBank database to confirm species identity. Sequence alignments were performed using Clustal_X version 2.1 [34] and were trimmed at both ends to remove low-quality regions. Molecular divergence and base substitution saturation analyses were performed using MEGA 6.0 [35] and DAMBE7 software [36]. Sequence gaps were treated as missing data. The evolutionary model best fitting the datasets was determined using Modeltest version 3.7 [37] based on the Akaike Information Criterion [38]. Phylogenetic trees were visualized using FigTree version 1.4 [39], and posterior probability values were calculated to assess the support for branching patterns.

### 2.2. Cultivation of Diatoms Under Different Nitrogen Sources

Two 1 L Erlenmeyer flasks containing filtered seawater supplemented with the Conway medium were used to cultivate the primary stock cultures. The flasks were sterilized, cooled, and inoculated with pure cultures of *Chaetoceros calcitrans* (15 × 10^4^ cells/mL) and *Thalassiosira weissflogii* (3 × 10^4^ cells/mL). Continuous illumination was provided by white fluorescent tubes, maintaining a light intensity of 3000–3500 lux. All treatments were conducted in duplicate using 1 L Erlenmeyer flasks. Four nitrogen source treatments were tested to maintain subcultures of *C. calcitrans* and *T. weissflogii* with nitrate (KNO_3_) as the control treatment, urea (N-Ur; T1), ammonium sulfate (N-Sulfate; T2), and ammonium nitrate (N-NH_3_; T3). Each nitrogen source was supplied at a final concentration of 1 mM, maintaining an N:P atomic ratio of 15:1 as in the original Conway medium. The specific nitrogen additions were potassium nitrate (KNO_3_) at 1147 μM, ammonium nitrate (NH_4_NO_3_) at 1000 μM, ammonium sulfate [(NH_4_)_2_SO_4_] at 1000 μM, and urea [CO(NH_2_)_2_] at 1000 μM, while other medium components were consistent across treatments, as shown in Table 2. Cultures were grown under aeration in Conway medium, with 10% (100 mL) of pure *C. calcitrans* and *T. weissflogii* cultures inoculated into 1 L of filtered and autoclaved seawater with a salinity of 30 ppt for all treatments.

#### 2.2.1. Determination of Microalgal Growth Rate

Microalgal biomass concentration was determined by cell counting and dry weight measurement. Microalgae cells were counted microscopically using a hemocytometer, a specialized slide consisting of two chambers, each divided into nine large squares (1.0 mm × 1.0 mm, or 1 mm^2^). The central counting area comprises 25 large squares, each further subdivided into 16 smaller squares. Cell density (D, cells/mL) was calculated using the following equation: D (cells/mL) = total number of cells counted/10 × 4 × 10^−6^, where 10 represents the number of squares in the two chambers, and 4 × 10^−6^ is the volume of the sample over one small square (0.004 mm^3^ or 0.2 mm × 0.2 mm × 0.1 mm), expressed in cm^3^ (mL). Cell density was measured every 24 h, and after 7 days of culture, protein, and carbohydrate contents were analyzed.

A defined volume of algal suspension (20 mL) was centrifuged at 3000 rpm for 10 min, and the precipitated cells were washed twice with distilled water. The biomass was transferred to a pre-weighed, dry filter paper and dried overnight in an oven at 60 °C until a constant weight was achieved [40,41]. Diatoms were collected by filtering cultures through 16 µm nylon net filters, which trapped the cells while allowing liquid to pass. The concentrated cells were subsequently dried at 50 °C for nutritional analysis.

#### 2.2.2. Chemical Analysis of Microalgae

Microalgal samples from each treatment were analyzed for moisture, crude protein, total lipids, crude fiber, ash, and fatty acid composition using standard methods outlined by the Association of Official Analytical Chemists [42].

### 2.3. Evaluation of Diatom-Based Diets for Litopenaeus Vannamei Larvae

*Litopenaeus vannamei* larvae were sourced from the El-Ekhlas hatchery in the Damietta protectorate. The shrimp were stocked in fiberglass tanks equipped with aeration. During the experiment, the average water salinity concentration was 30–31 ppt, the pH was 7.8, and the temperature ranged between 29 and 30 °C. The dissolved oxygen level averaged 6.8 mg/L, while ammonia concentrations averaged 0.05 mg/L. Water exchange was conducted at a rate of 50% per day, and aeration was provided using an oxygen water blower. The experiment was designed with three treatments, each conducted in triplicate. Nine 150 L fiberglass vessels, each containing 130–150 L of saltwater of consistent quality and conditions, were stocked at a density of 300 larvae per liter. The treatment groups included the following: *Chaetoceros calcitrans* at 50 × 10^4^ cells/mL as the ultimate density in the rearing tank, *Thalassiosira weissflogii* at 10 × 10^4^ cells/mL, and a combination of *C. calcitrans* and *T. weissflogii* at 30 × 10^4^ cells/mL and 5 × 10^4^ cells/mL, respectively. During the first two days of the experiment, the larvae were fed exclusively with diatoms twice daily. From the third day until the end of the experiment, feeding was supplemented with a powdered artificial fish feed containing 40% protein, administered twice daily alongside diatoms. The larvae were incubated in these conditions for three days, corresponding to the Zoea 1 to Mysis 1 phases, with diatoms provided as described above.

Diatoms were added as needed to maintain the initial density levels, which were measured four times daily using a hemocytometer and a light microscope. The density of each diatom species was monitored and recorded at time = 0, and the larvae were checked every 4–6 h following standard hatchery protocols. At the end of each 4–6 h interval, the diatom density was re-evaluated, and the difference from the initial density was calculated to estimate the number of diatom cells consumed by the larvae during the period. After the experiment, shrimp larvae were fasted for 24 h before their weight, length, and survival rate were measured.

### 2.4. Growth Measurements of Shrimp Larvae

The feeding rate of shrimp larvae at each stage, from Zoea 1 to Zoea 3, was determined by recording the total number of microalgae cells consumed using a hemocytometer. Mortality rates at each stage were calculated using the formula:

Mortality rate (%) = 100 × (number of dead shrimp)/(initial number of shrimp)(1)

The weight of shrimp larvae was measured on the final day of the experiment (Mysis 3) using a digital scale with 0.001 g accuracy. The length of larvae at each stage was determined using a microscope equipped with a calibrated slide.

### 2.5. Statistical Analysis

Data were analyzed using IBM SPSS software package version 20.0 (IBM Corp., Armonk, NY, USA). The Shapiro–Wilk test was employed to verify the normality of data distribution. Quantitative data were described using means and standard errors. Statistical significance was judged at the 5% level. For normally distributed quantitative variables, comparisons among groups were conducted using one-way ANOVA followed by Tukey’s post hoc test for pairwise comparisons.

## 3. Results

### 3.1. Morphological Identification of the Microalgae Used

**Figure 2:**
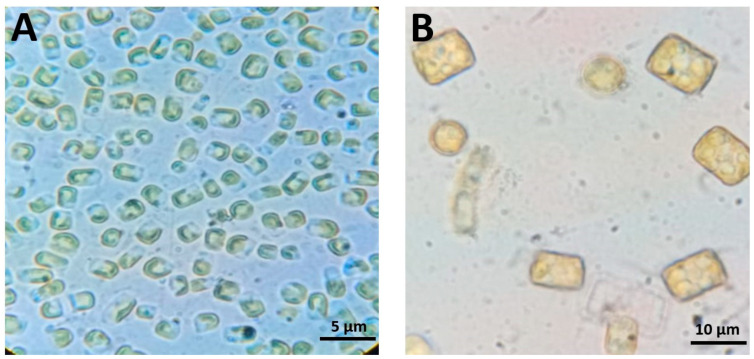
Microscopic images of *Chaetoceros* sp. (**A**) and *Thalassiosira* sp. (**B**).

Morphological identification of the diatoms *Chaetoceros* and *Thalassiosira* sp., obtained from the El-Ekhlas hatchery in Damietta Governorate (Mediterranean Sea), is presented in Figure 2. *Chaetoceros* cells form filamentous colonies, with adjacent valves connected by twisted setae extending from the poles of each valve. The valves and setae are lightly silicified. The valves are elliptic, and dissociated frustules result in neighboring valves remaining connected face-to-face. The valve face of the intercalary cells is slightly convex (Figure 2).

*Thalassiosira* sp. cells are short cylinders, varying in size from 4 to 32 µm in diameter. The cells tend to be larger in winter (15 µm) and smaller in summer (5 µm). These diatoms occur both singly and in groups, sometimes embedded in a gelatinous matrix. Their valves are round, flat, and have short mantles. The frustules are relatively lightly silicified, with fine areolae whose structural details are not visible under light microscopy (Figure 2).

### 3.2. Molecular Identification of Microalgal Species

**Figure 3:**
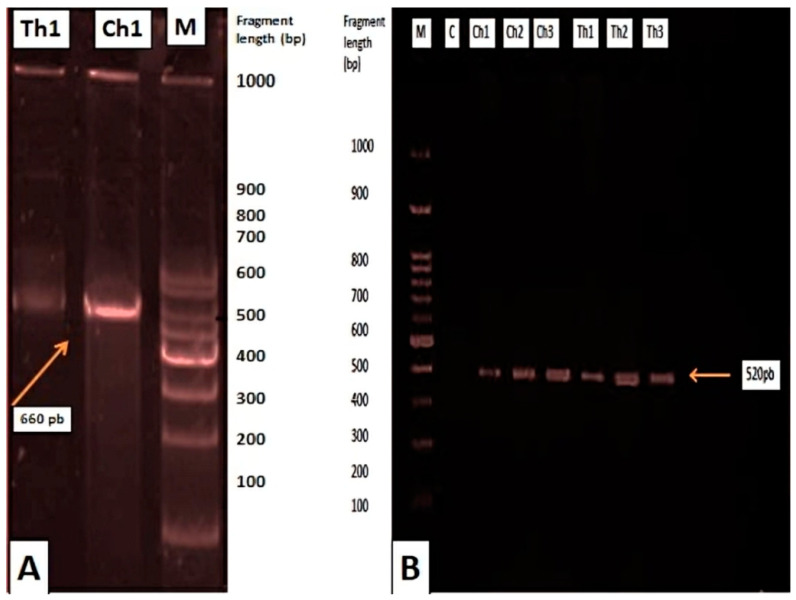
Specific genomic amplification products. (**A**) PCR product for the *rbcL* gene (~660 bp) of *Chaetoceros calcitrans* (Ch1) and *Thalassiosira weissflogii* (Th1), with the molecular size standard (M). (**B**) PCR product for the *ITS* region.

The details of amplification and sequencing are summarized in Table 1. Amplification of all three genes was highly successful. PCR products of *rbcL* were sequenced directly, whereas several amplified fragments of the 18S rRNA gene and *ITS* were sequenced after cloning. As shown in Figure 3, successful amplification of the *rbcL* gene (~660 bp) and *ITS* region was confirmed, with clear bands corresponding to the expected product sizes for *Chaetoceros calcitrans* and *Thalassiosira weissflogii*. The *rbcL* gene exhibited the highest BLAST hit success (four sequences; 99.2%), followed by *ITS* (two sequences; 74.1%). The *rbcL* sequences of *Chaetoceros* spp. and *Thalassiosira* spp. matched corresponding sequences in the families *Heterokontophyta–Bacillariales* (*Chaetoceros calcitrans*) and *Bacillariophyceae* (*Thalassiosira weissflogii*), respectively, as there were no existing *rbcL* sequences for these genera in the database. The *rbcL* sequences of *Chaetoceros calcitrans* and *Thalassiosira weissflogii* did not match sequences from other genera.

**Figure 4:**
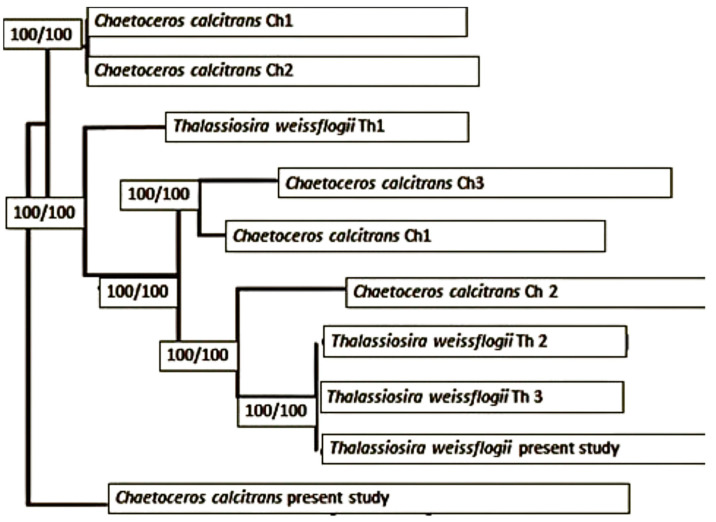
Phylogenetic analysis based on *rbcL* and *ITS-2* genes showing well-clustered clades of *Chaetoceros* and *Thalassiosira*, grouped within the *Bacillariales* clade. Bootstrap values (100/100) at the nodes represent strong support from Maximum Likelihood (ML) and Bayesian Inference (BI) analyses, respectively.

In total, 16 sequences of *ITS* and 16 sequences of *rbcL* were used for phylogenetic analysis. Based on *ITS* and *rbcL* gene sequences (Figure 3), the 578 sequences clustered into three main clades, including the ‘clade Bacillariophyceae’. The PCR products illustrated in Figure 3 provided the basis for constructing these phylogenies. The *rbcL* region failed to resolve diatom clustering relationships effectively compared to the *ITS* gene tree (Figure 4). In the *ITS* phylogeny (Figure 4), sequences from members of *Chaetoceros* and *Thalassiosira* formed a distinct ‘clade Thalassiosirales,’ with species from these genera separating clearly within the clade. However, other centric and pennate diatoms were clustered out of order. These findings suggest that the *ITS* region is more suitable for species discrimination and phylogenetic analyses of lower taxa within the *Thalassiosirales*.

### 3.3. Effect of Nitrogen Sources on Growth Rates of Diatoms

Table 3 highlights variations in the dry weight of *C. calcitrans* biomass across treatments during the incubation period. The growth rates of *C. calcitrans* showed significant differences (*p* < 0.05) among treatments with different nitrogen sources from day 2 to day 6. The highest cell count was recorded in T3, reaching 4.60 × 10^6^ cells mL^−1^ on day 6, while the lowest count was observed in the control group, with 3.24 × 10^6^ cells mL^−1^ on the same day. The minimum dry weight was 21.05 mg mL^−1^ in the control group on day 4, while the maximum was 22.7 mg mL^−1^ in T3 on day 6 (Table 3).

The growth characteristics of *T. weissflogii* in Table 4 shows the effects of nitrogen sources on the growth of *T. weissflogii*, revealing significant differences (*p* < 0.05) among treatments from day 2 to day 6. The control group exhibited the highest cell count, reaching 0.322 × 10^6^ cells mL^−1^ on day 4, with no significant difference compared to T1 (0.318 × 10^6^ cells mL^−1^). The lowest count was observed in T3 (0.108 × 10^6^ cells mL^−1^) on day 4. The trend in cell counts was mirrored in the dry weight measurements. The lowest dry weight (10.25 mg mL^−1^) was found in T2 on day 4, while the highest (12.75 mg mL^−1^) was observed in the control group, closely followed by T3 (12.70 mg mL^−1^) on day 6 (Table 4).

### 3.4. Effect of Nitrogen Sources on Chemical Composition of Diatoms

Table 5 summarizes the chemical composition of *C. calcitrans* and *T. weissflogii* under varying nitrogen sources. The nitrogen source significantly influenced the chemical composition of *C. calcitrans* compared to the control group. The protein content was significantly higher in T1, T2, and T3 compared to the control, with the greatest increase observed in T3. Similarly, lipid content was significantly elevated in all treatments relative to the control, with T2 and T3 showing more pronounced differences. In contrast, carbohydrate content decreased significantly in all treatments compared to the control, with the largest reduction observed in T3. No significant differences were detected in moisture and ash content among the treatments. For *T. weissflogii*, all treatments showed significantly higher protein content compared to the control, with T3 displaying the most substantial improvement (Table 5). Lipid content also increased significantly in all treatments relative to the control, with T3 exhibiting the greatest enhancement. Conversely, carbohydrate content was significantly reduced in all treatments compared to the control, with T3 showing the largest decrease. Moisture and ash content did not differ significantly between treatments and the control (Table 5).

### 3.5. Feeding Rate by White Leg Shrimp (Litopenaeus vannamei) Larvae

**Figure 5:**
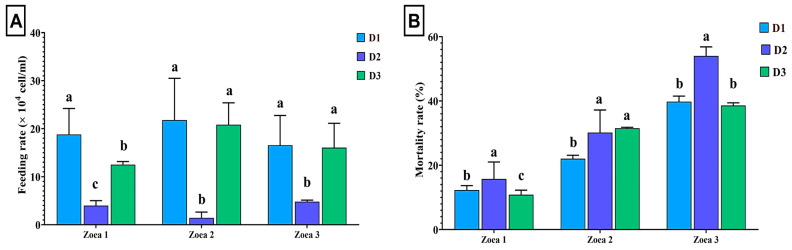
Feeding rate (**A**) and mortality rate (**B**) of *L. vannamei* fed on *C. calcitrans* (D1), *T. weissflogii* (D2), and or their mixture (D3) across different growth phases of the shrimp. Different small letters above the error bar indicate significant differences at *p* < 0.05.

As shown in Figure 5, the feeding rate of microalgae cells varied among treatments and growth phases of shrimp (*L. vannamei*). In the Zoea 1 phase, shrimp fed on *C. calcitrans* (D1) exhibited a higher feeding rate compared to those fed on *T. weissflogii* (D2) or the mixed diet (D3). During the Zoea 2 phase, the shrimp that fed on D1 maintained the highest feeding rate, while those that fed on D2 showed the lowest feeding rate. In the Zoea 3 phase, the feeding rate of the shrimp that fed on D1 remained higher than those that fed on D2 but comparable to the mixed diet (Figure 5).

### 3.6. Mortality Rate for Shrimp Larvae Treated with Diatoms

Mortality rates were significantly affected by the diet across all growth phases (Figure 5). In the Zoea 1 phase, the shrimp that fed on *T. weissflogii* (D2) had a significantly higher survival rate compared to those that fed on the mixed diet (D3) or *C. calcitrans* (D1). During the Zoea 2 phase, mortality rates were significantly higher in the shrimp that fed on T2 and T3 compared to T1. In the Zoea 3 phase, shrimp fed on T2 exhibited the highest mortality rate, while no significant differences were observed between shrimp fed on T1 and T3.

### 3.7. Growth Performance of L. vannamei Larvae

The body length of *L. vannamei* larvae was significantly influenced by diet across most growth phases (Table 6). In the Zoea 1 phase, the larvae that fed on the mixed diet (D3) exhibited a significantly greater body length compared to those fed on *T. weissflogii* (D2) or *C. calcitrans* (D1) (*p* < 0.001). During the Zoea 2 phase, larvae in the D3 group showed the greatest body length, significantly surpassing those in the D1 group, while no significant difference was observed between the D2 and D3 groups (*p* = 0.042). In the Zoea 3 phase, no significant differences in body length were detected among the dietary treatments (*p* = 0.104), although larvae in the D3 group maintained slightly higher values compared to the other groups. In the Mysis 1 phase, the larvae that fed on the D2 and D3 diets achieved significantly greater body lengths than those in the D1 group (*p* = 0.006). During the Mysis 2 and Mysis 3 phases, larvae in the D3 group consistently had the highest body lengths, significantly exceeding those in the D2 and D1 groups. In the Mysis 2 phase, larvae fed the D3 diet were significantly larger than those in the D1 and D2 groups (*p* < 0.001). Similarly, in the Mysis 3 phase, the larvae that fed on the D3 diet were significantly larger than those in the D1 and D2 groups (*p* = 0.011).

## 4. Discussion

Nitrogen availability plays a crucial role in determining the growth and biomass production of microalgae, with the impact varying by nitrogen type and microalgal species [43]. This study found that urea resulted in the highest values, followed by ammonium nitrate and ammonium sulfate (cost-effective nitrogen sources), while potassium nitrate yielded the lowest dry weight. Ammonium is recognized as essential for microalgae, surpassing the importance of nitrates or nitrites, due to its status as a condensed form of nitrogen [28]. Ammonium is directly assimilated with amino acids within the cell, whereas nitrates or nitrites require reduction to ammonium prior to utilization [29]. Our findings align with previous studies showing that ammonium-based nitrogen sources enhance biomass production during the stationary phase [44] and that urea significantly increases cell densities in microalgal cultures [45,46]. Additionally, a surplus of nitrogen (NH_4_ 25:1) resulted in greater dry-weight production overall, as it increased maximum cell densities [44]. This aligns with our findings, where treatments with urea, ammonium nitrate, and ammonium sulfate resulted in significantly higher dry weights compared to the control with potassium nitrate.

The observed higher protein and lipid content for both *Chaetoceros calcitrans* and *Thalassiosira weissflogii* in the ammonium treatment can be attributed to the efficient assimilation of ammonium, which is directly incorporated into amino acids and lipids, supporting faster growth and higher biomolecule accumulation [29]. In contrast, the control (nitrate) resulted in the highest carbohydrate content because nitrate assimilation is linked to increased carbon partitioning into storage carbohydrates, as seen in *Chlamydomonas reinhardtii*, where nitrate enhances starch and triacylglycerol accumulation [47]. Carbohydrate production varies with growth phases; nitrate enhances production during both exponential and stationary phases, while ammonium may inhibit carbohydrate synthesis [48]. Khwancharoen et al. [28] found that *Amphora coffeaeformis* cultured with ammonium sulfate had the highest protein content, but the difference was not significant compared to other treatments. However, *A. coffeaeformis* cultured with nitrates had the highest lipid content, as well as saturated and monounsaturated fatty acids. Simsek and Cetin [49] studied *Chlorella vulgaris* using ammonium sulfate and sodium nitrate as nitrogen sources and found that these treatments resulted in a high concentration of lipid content, with ammonium nitrate yielding the greatest increase in protein content. Similarly, Sánchez-García et al. [50] found that nitrates influenced biomass production and lipid composition in species like *Monoraphidium contortum*, *Tetraselmis suecica*, and *Chlorella minutissima*.

In this study, the results showed significant differences in the cell count of *T. weissflogii* across all treatments throughout the incubation period. The highest cell count was observed in the control treatment on the fourth day, while the lowest was recorded in the T3 treatment on the same day. These findings are consistent with those of Lourenco et al. [51], who found that species such as *Hillea* sp. and *Proroeentrum minimum* failed to grow with ammonium-N due to the toxic effects of high ammonia concentrations. Similarly, other species, including *Hillea* sp. and *Nannoehloropsis oculata* on urea-N, *Isochrysis galbana*, *Phaeodactylum tricornutum*, and *Synechococcus subsalsus* on ammonium-N, showed significantly lower final yields compared to other nitrogen sources. Fernández-Herrera et al. [52] also reported that *T. weissflogii* cultivated with NH_4_Cl produced higher yields of EPA and DHA, offering a better yield–cost ratio for biomass and lipid production. Moreover, treatments with NaNO_3_ and NH_4_Cl resulted in peak chlorophyll *a* values earlier in the growth cycle, while higher concentrations (890 µM) led to greater chlorophyll *a* production at peak values.

Herein, the chemical composition of *T. weissflogii* showed that the highest protein content was found in T3, while the lowest was in T2. For lipids, the highest percentages were observed in T3, followed by T2 and T1, with the lowest percentages observed in the control. In terms of carbohydrates, the control had the highest percentage, while the lowest was recorded in T3. As previously described, some agriculture fertilizers worked well or may be better for *T. weissflogii*, while others showed a negative response by the microalgae in terms of cell density and chemical composition [49,51,52].

The current study found that introducing a mixed diatom diet for *L. vannamei* larvae results in significant improvements, including lower mortality and higher growth rates, compared to diets consisting of individual diatom species. Similarly to this finding, Panjaitan et al. (2015) found that survival rates for *C. calcitrans* and *T. weissflogii* were improved, with the mixed diet yielding the highest survival rate. Also, Tam et al. [21] reported that adding living *T. weissflogii* biomass to the diet of white-leg shrimp larvae at the nauplii 6 stage resulted in increased body length, weight, and survival rates. In line with Pérez-Morales et al. [53], who found that *Chaetoceros* resulted in the highest survival rate (66.63%) and growth (average length of 3.17 mm) in *L. vannamei* larvae, our study showed significant differences in the survival rates of *L. vannamei* that were fed different diets. Muller-Feuga [54] suggested that approximately 1 m^3^ of microalgal culture containing 3 × 10^6^ cells or 65 g of dry weight is required to generate 10^6^ post-larvae. Based on our findings, we recommend providing approximately 40 × 10^4^ cells/mL of *C. calcitrans* and 15 × 10^4^ cells/mL of *T. weissflogii* to meet the nutritional needs of the larvae.

The improved growth and lowered mortality of *L. vannamei* larvae that were fed a mixed diatom diet can be attributed to the distinct nutritional profiles of *C. calcitrans* and *T. weissflogii*. *T. weissflogii* is known for its higher levels of protein, total fatty acids, and polyunsaturated fatty acids, including essential nutrients like eicosapentaenoic acid (EPA) and docosahexaenoic acid (DHA), which are critical for shrimp larval growth [55]. These nutrients play a pivotal role in enhancing the survival and metamorphosis rates of shrimp larvae, particularly during early growth stages. Moreover, *T. weissflogii* demonstrated superior antimicrobial properties compared to other microalgae, which may further contribute to the improved survival and growth of *L. vannamei* larvae [56]. On the other hand, *C. calcitrans*, while commonly used in shrimp larviculture, has a relatively lower nutritional profile, especially in terms of essential fatty acids [53]. The combination of these two diatoms in a mixed diet appears to provide a more balanced nutritional composition, ensuring that larvae receive a broader spectrum of essential nutrients, which likely contributes to the improved survival and growth observed in the mixed diet treatment. The synergistic effects of these two diatoms may enhance larval development by meeting the diverse nutritional requirements of the larvae during critical growth periods.

The findings of this study have significant practical implications for commercial shrimp hatcheries. Optimizing nitrogen sources in microalgal cultures can improve shrimp larvae growth and survival rates. For instance, cost-effective ammonium-based nitrogen sources (urea, ammonium sulfate, and ammonium nitrate) significantly increased protein (up to 41.93% in *T. weissflogii*) and lipid content (up to 25.47% in *T. weissflogii*), while traditional nitrate-based sources provided higher carbohydrate content (up to 37.04% in *C. calcitrans*). Mixed diatom diets, combining *C. calcitrans* and *T. weissflogii*, provided a balanced nutritional profile, enhancing shrimp larval performance, as evidenced by increased body length (up to 1.588 mm in Mysis 3) and improved survival rates, thereby boosting hatchery productivity and profitability. However, this study focused on a limited number of microalgal species and nitrogen sources; expanding to include a wider range could provide a more comprehensive understanding. Furthermore, the long-term effects of nitrogen-optimized microalgal diets on the entire lifecycle of *L. vannamei*, from larvae to adulthood, were not explored and should be investigated to fully assess their potential benefits for aquaculture.

## 5. Conclusions

Based on the study’s findings, using cost-effective nitrogen sources such as urea, ammonium sulfate, and ammonium nitrate in the cultivation of *C. calcitrans* and *T. weissflogii* significantly improved microalgal growth, protein, and lipid content while reducing carbohydrates. A mixed diet of these diatoms further enhanced growth performance and reduced mortality in *L. vannamei* larvae, offering a sustainable and cost-effective approach for shrimp aquaculture. Future studies should investigate the scalability of this approach, its economic impacts on commercial shrimp hatcheries, and potential interactions with other aquaculture systems.

## Figures and Tables

**Table 1:** Sequences of primers used in this study.

Primer	Sequence (5’–3’)	Approx. Fragment Length (bp)
*ITS*-F	CSMACAACGATGAAGRRCRCAGC	520
*ITS*-R	TCCCDSTTCRBTCGCCVTTACT	
*rbcL*-F	ATGTCTCAATCTGTAWCAGAACGGACTC	660
*rbcL*-R	TAARAAWCKYTCTCTCCAACGCA	

*ITS*, internal transcribed spacer; *rbcL*, ribulose-bisphosphate carboxylase.

**Table 2:** Nitrogen sources used for culturing *C. calcitrans* and *T. weissflogii*, along with their price values.

Nitrogen Source	Chemical Formula	Molarity (g/mol)	Nitrogen Content (%)	Density (g/cm^3^)	Nitrogen (g/mol)	Price (US$/kg) *
Potassium nitrate (control)	KNO_3_	101.1	13.4	2.11	13.54	100
Urea (T1)	CO(NH_2_)_2_	60.06	46	1.32	27.62	0.30
Ammonium sulfate (T2)	(NH_4_)_2_SO_4_	132.1	21	1.77	27.74	0.20
Ammonium nitrate (T3)	NH_4_NO_3_	80.04	33.5	1.72	26.81	0.30

* Prices are based on the local market, with an approximate exchange rate of 1 US$ ≈ 50 Egyptian pounds.

**Table 3:** Effect of different nitrogen sources on growth parameters of *C. calcitrans*. Cell count (×10^6^ cells/mL) and dry weight biomass (mg/mL) over the incubation period.

Growth Parameters	Incubation Time (days)	Treatments *				*p* Value
		Control	T1	T2	T3	
Cell Count	Day 0	0.17 ^a^ ± 0.01	0.11 ^a^ ± 0.01	0.14 ^a^ ± 0.02	0.18 ^a^ ± 0.03	0.090
	Day 2	1.45 ^b^ ± 0.20	1.49 ^b^ ± 0.14	2.28 ^a^ ± 0.17	1.57 ^b^ ± 0.17	0.006
	Day 4	3.13 ^b^ ± 0.36	3.56 ^ab^ ± 0.27	2.91 ^c^ ± 0.54	3.86 ^a^ ± 0.13	0.256
	Day 6	3.24 ^b^ ± 0.46	3.17 ^b^ ± 0.34	4.28 ^ab^ ± 0.56	4.60 ^a^ ± 0.54	0.101
Dry weight	Day 0	10.70 ^a^ ± 0.20	10.60 ^a^ ± 0.40	10.35 ^a^ ± 0.15	10.30 ^a^ ± 0.10	0.627
	Day 2	13.70 ^c^ ± 1.0	15.65 ^a^ ± 1.25	13.45 ^c^ ± 0.35	14.65 ^b^ ± 0.15	0.346
	Day 4	21.05 ^c^ ± 0.05	23.05 ^a^ ± 0.05	15.25 ^d^ ± 0.25	22.10 ^b^ ± 0.10	<0.001
	Day 6	20.35 ^b^ ± 0.05	18.70 ^c^ ± 0.20	21.60 ^b^ ± 0.10	22.70 ^a^ ± 0.10	<0.001

* The treatments were based on four nitrogen sources: nitrate (KNO_3_) as the control, urea (T1), ammonium sulfate (T2), and ammonium nitrate (T3). Means in the same row having the same superscript letters are not significantly different (*p* < 0.05).

**Table 4:** Effect of different nitrogen sources on growth parameters of *T. weissflogii*, cell count (×10^6^ cells/mL) and dry weight biomass (mg/mL), over the incubation period.

Growth Parameters	Incubation Time (days)	Treatments *				*p* Value
		Control	T1	T2	T3	
Cell Count	Day 0	0.017 ^a^ ± 0.002	0.021 ^a^ ± 0.003	0.022 ^a^ ± 0.004	0.015 ^a^ ± 0.003	0.362
	Day 2	0.070 ^b^ ± 0.009	0.130 ^a^ ± 0.012	0.041 ^c^ ± 0.005	0.077 ^b^ ±0.017	<0.001
	Day 4	0.322 ^a^ ± 0.019	0.318 ^a^ ± 0.033	0.210 ^b^ ± 0.026	0.108 ^c^ ± 0.028	<0.001
	Day 6	0.232 ^ab^ ± 0.032	0.162 ^b^ ± 0.036	0.260 ^a^ ± 0.014	0.062 ^c^ ± 0.015	<0.001
Dry weight	Day 0	9.55 ± 0.15	9.30 ± 0.20	9.35 ± 0.15	9.65 ± 0.15	0.474
	Day 2	10.90 ± 0.90	10.95 ± 0.05	10.25 ± 0.05	10.70 ± 0.40	0.751
	Day 4	12.75 ^a^ ± 0.05	10.25 ^c^ ± 0.05	10.25 ^c^ ± 0.05	12.05 ^b^ ± 0.05	<0.001
	Day 6	12.75 ^a^ ± 0.05	10.23 ^b^± 0.20	10.20 ^b^ ± 0.10	12.70 ^a^ ± 0.10	<0.001

* The treatments were based on four nitrogen sources: nitrate (KNO_3_) as the control, urea (T1), ammonium sulfate (T2), and ammonium nitrate (T3). Means in the same row having the same superscript letters are not significantly different (*p* < 0.05).

**Table 5:** Chemical composition of *Chaetoceros calcitrans* and *Thalassiosira weissflogii* at different nitrogen sources.

Parameters	Treatments *				*p* Value
	Control	T1	T2	T3	
*Chaetoceros calcitrans*					
Moisture %	7.62 ± 0.14	7.65 ± 0.08	7.63 ± 0.05	7.49 ± 0.08	0.621
Protein %	30.40 ^c^ ± 0.44	33.67 ^b^ ± 0.47	32.87 ^bc^ ± 0.33	37.03 ^a^ ± 0.34	<0.001
Lipids %	13.77 ^c^ ± 0.20	17.47 ^b^ ± 0.66	20.37 ^a^ ± 0.52	21.40 ^a^ ± 0.42	<0.001
Ash %	11.18 ± 0.03	11.0 ± 0.13	11.27 ± 0.21	11.05 ± 0.11	0.515
Carbohydrates %	37.04 ^a^ ± 0.41	30.22 ^b^ ± 0.18	27.87^c^ ± 0.66	23.03 ^d^ ±0.43	<0.001
*Thalassiosira weissflogii*					
Moisture %	7.54 ^a^ ± 0.05	7.65 ^a^ ± 0.10	7.60 ^a^ ± 0.08	7.32 ^a^ ± 0.03	0.046
Protein %	33.60 ^c^ ± 0.44	37.73 ^b^ ± 0.24	31.60 ^d^ ± 0.58	41.93 ^a^ ± 0.75	<0.001
Lipids %	17.60 ^c^ ± 0.44	21.60 ^b^ ± 0.51	21.83 ^b^ ± 0.20	25.47 ^a^ ± 0.55	<0.001
Ash %	10.87 ± 0.18	11.12 ± 0.17	11.10 ± 0.12	11.09 ± 0.27	0.779
Carbohydrates %	30.39 ^a^ ± 0.32	21.90 ^c^ ± 0.67	27.87 ^b^ ± 0.56	14.19 ^d^ ± 0.50	<0.001

* The treatments were based on four nitrogen sources: nitrate (KNO_3_) as the control, urea (T1), ammonium sulfate (T2), and ammonium nitrate (T3). Means in the same row having the same superscript letters are not significantly different (*p* < 0.05).

**Table 6:** Changes in *L. vannamei* body length (mm) with different diatoms diets across different growth phases of the shrimp.

Growth Phases	Experimental Groups			*p* Value
	D1	D2	D3	
Zoea 1	0.354 ^c^ ± 0.001	0.378 ^b^ ± 0.0	0.407 ^a^ ± 0.005	<0.001
Zoea 2	0.646 ^b^ ± 0.006	0.693 ^ab^ ± 0.006	0.715 ^a^ ± 0.024	0.042
Zoea 3	0.900 ± 0.019	0.928 ± 0.002	0.963 ± 0.023	0.104
Mysis 1	1.035 ^b^ ± 0.025	1.207 ^a^ ± 0.031	1.230 ^a^ ± 0.031	0.006
Mysis 2	1.140 ^c^ ± 0.006	1.365 ^b^ ± 0.032	1.475 ^a^ ± 0.014	<0.001
Mysis 3	1.323 ^c^ ± 0.025	1.413 ^b^ ± 0.022	1.588 ^a^ ± 0.065	0.011

The means in the same row that have the same superscript letters are not significantly different (*p* < 0.05). The experimental groups included white-leg shrimp larvae (*Litopenaeus vannamei*), which were fed diets of *C. calcitrans* (D1), *T. weissflogii* (D2), or a combination of both microalgae (D3).

## Data Availability

Data will be made available on request.
